# Understanding Health Care Provider Burnout When Caring for Patients With Refractory Epilepsy in the United States

**DOI:** 10.1212/CPJ.0000000000200260

**Published:** 2024-02-13

**Authors:** Mei Lu, Enrico Speri, A. Lauks, Charles Leibson, Satish C. Rao

**Affiliations:** Takeda Pharmaceuticals USA, Inc. (ML, SCR), Lexington, MA; and IQVIA (ES, AL, CL), Cambridge, MA.

## Abstract

**Background and Objectives:**

Among health care providers (HCPs), neurologists have one of the highest rates of burnout in the United States, compromising the quality and accessibility of patient care. Patients with refractory epilepsy are especially challenging to treat. This study aims to understand the burnout level in neurologists treating patients with refractory epilepsy and identify key contributing factors.

**Methods:**

US board-certified pediatric/adult neurologists who devote ≥50% of their time to clinical practice and treat ≥10 unique patients with refractory epilepsy annually were invited to take a noninterventional quantitative survey, designed to capture key elements of the HCP's background, burnout level, current practice, burden domains, and satisfaction with current antiseizure medications (ASMs). Burnout in 3 domains (emotional exhaustion, depersonalization, and personal accomplishment) was assessed by the validated Maslach Burnout Inventory–Human Services Survey.

**Results:**

From March 11, 2022, to April 10, 2022, a total of 138 neurology-specialist HCPs participated in the survey, divided between adult epileptologists (n = 44), adult neurologists (n = 41), pediatric epileptologists (n = 36), and pediatric neurologists (n = 17). Of participating HCPs, 61% experienced at least some burnout (≥1 of 3 burnout domains categorized as *high*), and 4% experienced high burnout (3 of 3 burnout domains categorized as *high*). High burnout levels were driven by high pediatric and inpatient caseloads and unexpected pediatric patient reluctance to transition to adult care. HCPs with high burnout had a higher yearly caseload of patients with refractory epilepsy. Most HCPs (approximately 90%) indicated that patients with refractory epilepsy were more difficult to manage than those with nonrefractory epilepsy. The proportion of HCPs satisfied or extremely satisfied with ASMs was lower for patients with refractory epilepsy (20%) than that for patients with nonrefractory epilepsy (73%). Dissatisfaction was mostly due to workload and latency of the insurance approval process, out-of-pocket costs, and poor efficacy, safety, and tolerability. For 32% of HCPs, stopping practicing or moving to another practice within 5 years was probable or very probable.

**Discussion:**

Some burnout is common among HCPs who treat patients with refractory epilepsy. However, management of refractory epilepsy is challenging, and satisfaction with available ASMs is low. Thus, addressing these contributing factors may help to alleviate HCP burnout.

## Introduction

High-quality and affordable health care depends on the accessibility and well-being of health care providers (HCPs).^1^ Burnout, however, can affect the well-being of HCPs and quality of patient care. Conceptualized as a symptom resulting from unmanaged chronic work-related stress, burnout is characterized by 3 domains: emotional exhaustion (EE), depersonalization (DP), and reduced personal accomplishment (PA).^2,3^ Burnout is more common among HCPs compared with the rest of the general workforce in the United States.^4,5^ HCPs experiencing burnout are more likely to have impaired clinical judgment and a lack of empathy, which can negatively affect patient care and satisfaction.^6-8^

Among HCP specialties in the United States, neurologists have one of the highest rates of burnout.^4,5^ Furthermore, neurologist burnout could worsen as the demand for care increases.^9^ The neurologist shortage is projected to worsen by 2025, which could markedly affect the care of patients and health care costs.^10^ Identified risk factors of burnout among neurologists include high patient caseload per week, hours worked, nights on call, number of outpatients seen, feelings of loss of autonomy, a high administrative burden, and an inadequate support staff.^6^ Existing studies, however, have not evaluated how treatment of specific disorders affects neurologist burnout.

Epilepsy is one of the most common neurologic diseases worldwide, with approximately 5 million people diagnosed annually.^11^ Approximately 30%–40% of patients with epilepsy have drug-resistant or refractory epilepsy.^12,13^ Drug resistance in epilepsy is defined as “failure of adequate trials of 2 tolerated and appropriately chosen and used” antiseizure medications (ASMs) “to achieve sustained seizure freedom.”^14^ Treatment of patients with refractory epilepsy is very challenging owing to the necessary management of psychosocial dysfunction and cognitive decline along with a high mortality rate and the limitations of currently available ASMs.^15-18^ Thus, there is a critical unmet need for effective and safe ASMs to treat patients with refractory epilepsy.^19^

This study identifies the burnout level and associated factors among HCPs who treat patients with refractory epilepsy in the United States. Furthermore, differences are described in managing patients with refractory vs nonrefractory epilepsy and HCP satisfaction with current ASMs.

## Methods

### Study Design and Data Collection

The study design for this noninterventional, cross-sectional, quantitative survey included 2 components: a survey pretest and the online survey. Survey pretests were conducted with at least 1 representative for each specialty (epileptologist vs neurologist) and 1 representative for each patient population served (adult vs pediatric). A total of 3 HCPs (2 pediatric epileptologists and 1 adult neurologist) were interviewed during 45-minute video conference calls. The survey pretest validated our final online survey across several dimensions before it was shared with a wider physician audience. Tested dimensions ensured that the survey worked as intended with clear flow, logic, and language and had appropriate inclusion criteria and accurate selection options.

The online survey consisted of 5 sections: a screening section of 13 questions to identify eligible respondents and gather consent, followed by sections designed to capture elements of HCPs' current practice (12 questions); challenges associated with treating patients with refractory vs nonrefractory epilepsy (8 questions); physician perception of ASM (3 questions); and domains of burden and levels of burnout/stress/depression (11 questions). Questions included the number of patients seen over the last year, overall caseload, level of difficulty in transitioning from pediatric to adult care, satisfaction and importance of ASM attributes, and other potential stressors or unmet needs in the management of their patients' treatment.

### Study Population

Between March 11, 2022, and April 10, 2022, 138 US pediatric and adult neurologists/epileptologists participated in the online survey. Recruitment of eligible HCPs was conducted through SurveyHealthcareGlobus, which has access to a network of approximately 19,000 neurologists. Eligible HCPs were board-certified neurologists, who devote >50% of their time to clinical practice vs other activities (e.g., research, teaching, and administration) and treat ≥10 unique patients with refractory epilepsy per year. Epileptologists are board-certified neurologists who have completed a fellowship in epilepsy. Pediatric neurologists/epileptologists treat patients with epilepsy, with ≥50% of patients younger than 18 years.

### Maslach Burnout Inventory–Human Services Survey

**Figure 1:**
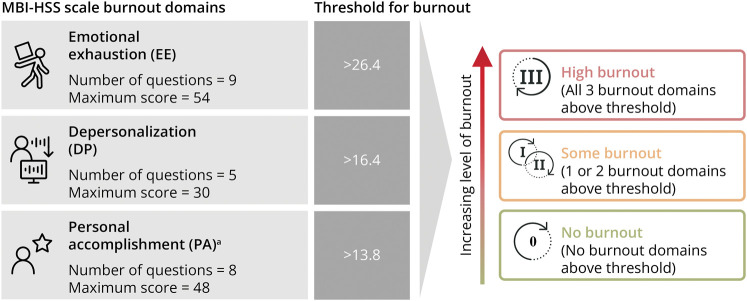
MBI-HSS Scale to Determine HCP Burnout Tier Physicians were assigned 1 of 3 burnout tiers (no burnout, some burnout, and high burnout) based on their scores for EE, DP, and PA. Each MBI-HSS question was answered on a 7-point scale (0 = never, 6 = every day).^2^ ^a^Indicates a reverse-scoring scale. DP = depersonalization; EE = emotional exhaustion; HCP = health care provider; MBI-HSS = Maslach Burnout Inventory–Human Services Survey; PA = personal accomplishment.

The Maslach Burnout Inventory–Human Services Survey (MBI-HSS) scale is a validated tool to evaluate physician burnout in 3 domains: EE, DP, and PA (Figure 1).^2,20,21^ Questions addressed HCP's mental health, stress levels and coping mechanisms, quality of life, and enjoyability of daily tasks. Physicians also rated their work autonomy, how meaningful their work is to them, and their well-being compared with other physicians.

This study used the equation-based threshold methodology recommended by the creators of the MBI-HSS scale. Burnout thresholds were calculated as follows: high EE is any score that is 0.5 times the SD over the mean score; high DP is any score that is 1.25 times the SD over the mean score; high PA is any score that is 0.1 SD over the mean score (accounting for a reversed PA scale). Mean scores and SD values of the current study population were used.

The MBI-HSS scale divided HCPs into 3 burnout tiers: no burnout, some burnout, and high burnout. “High burnout” was defined as scoring above the threshold in all 3 domains, whereas “some burnout” was defined as scoring above the threshold in 1 or 2 domains, and “no burnout” was defined as scoring below the threshold in all domains. Results compared the different burnout tiers with the overall study population, including all surveyed HCPs.

### Quality Control

This study relies on the respondents' truthful and accurate responses to survey questions. We examined internal respondents' answers to identify contradiction or “flat-lining” of responses (described as answering most questions with the same score) to limit false or inaccurate responses. In addition, to achieve our final sample size of 138 neurology-specialist HCPs, we removed respondents with inappropriately fast survey completion times (<10 minutes), which indicated little or poor consideration given to responses. Responses were normalized for each respondent, and mean, median, and SD values on the normalized scores were reported.

### Statistical Analysis

We conducted an analysis of burnout drivers to identify major survey variables related to physician burnout that would allow us to segment the physician sample. Among the multiple statistical methodologies tested, including Naïve Bayesian, Random Forest, or Boosted Tree, the recursive partitioning method was chosen as the most appropriate method given its ability to manage the nonlinear relationship between burnout levels and the variables in the survey and improved model interpretability. Recursive partitioning is a statistical method for multivariable analysis, which creates a decision tree that strives to correctly classify all survey physicians into 3 burnout tiers by splitting them into subpopulations based on the various survey variables (tree nodes). In this research, recursive partitioning used a nested tree approach to identify each burnout tier. A first tree identified the descriptors of the high burnout tier vs the remainder of the survey sample, while a second tree (nested tree) used the untiered sample to identify the descriptors for some vs no burnout tiers.^22^ The statistically significant (*p* values <0.05 in both the likelihood ratio test and the Pearson χ^2^ test) triads of descriptors identified were then leveraged to allocate HCPs to their respective burnout tier. The splitting of burnout groups was guided by G2 (likelihood ratio χ^2^).

### Standard Protocol Approvals, Registrations, and Patient Consents

All HCP respondents provided consent to the terms and conditions of the survey in this study. The WCG institutional review board granted an ethics review exemption for this study because the research included only interactions involving educational tests, survey procedures, interview procedures, or observations of public behavior; and there are adequate provisions to protect the privacy of participants and to maintain the confidentiality of data.

### Data Availability

Anonymized data not published within this article will be made available by request from any qualified investigator.

## Results

### Neurology-Specialist HCP Demographics

Between March 11, 2022, and April 10, 2022, 138 neurology-specialist HCPs who treated patients with refractory epilepsy participated in the survey, including 41 adult neurologists, 44 adult epileptologists, 17 pediatric neurologists, and 36 pediatric epileptologists (Table). Overall, most HCPs were men (68%), with an average age of 51 years and ≥ an average of 17 years of experience in practice.

**Table:** Neurology-Specialist HCP Demographics

Parameter	Adult neurologist	Adult epileptologist	Pediatric neurologist	Pediatric epileptologist	Overall
	(n = 41)	(n = 44)	(n = 17)	(n = 36)	(n = 138)
Age, y					
Mean (SD)	54 (10.4)	49 (10.0)	50 (13.4)	50 (8.5)	51 (10.3)
Years in practice					
Mean (SD)	20 (7.8)	16 (7.8)	18 (9.8)	17 (6.7)	17 (7.9)
Sex, %					
Female	22	23	35	42	29
Male	71	75	65	58	68
Prefer not to say	7	2	0	0	3
Work time split, %					
Clinical practice	94	93	91	88	92
Research	1	1	2	4	2
Teaching	2	3	4	5	3
Administration	2	3	2	4	3

Abbreviation: HCP, health care provider.

### Descriptors of Burnout

**Figure 2:**
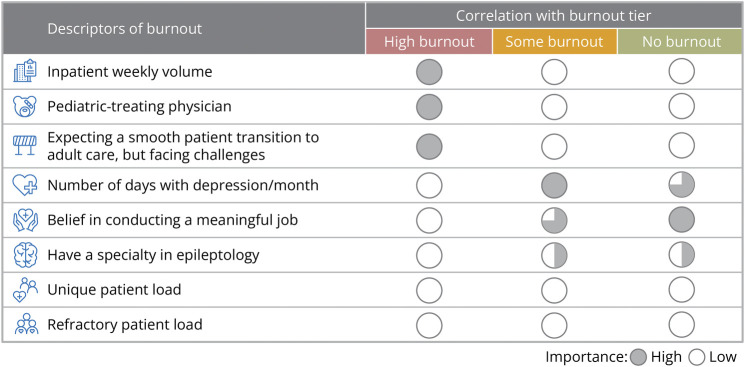
Health Care Provider Burnout Descriptors by Burnout Tier Circle shading indicates importance from high (fully shaded circle) to low (no shading). High burnout HCPs met all 3 descriptors of high burnout. HCPs with some or no burnout met ≥1 of the respective descriptors. HCP = health care provider.

Based on survey results (eTable 1, links.lww.com/CPJ/A511), we identified several burnout descriptors that, when combined, segmented HCP responses into 1 of the 3 burnout tiers: “no,” “some,” or “high” (Figure 2). Among HCPs who completed the survey (n = 138), 61% (n = 85) experienced at least some burnout, with 4% (n = 6) experiencing high burnout (eFigure 1, links.lww.com/CPJ/A508).

HCPs with high burnout (n = 6) were characterized by the following 3 factors combined: first, they managed a high caseload of pediatric patients compared with adult patients. HCPs with high burnout treated, on average, 60% pediatric patients, whereas the overall average for pediatric patients was 35%. Second, they treated ≥28 inpatients each week. The average number of inpatients per week reported by HCPs with high burnout was 42, vs 14 overall. Third, this subset of HCPs expected a smooth transition from pediatric to adult care, but experienced unexpected patient reluctance to transition. None of the HCPs with high burnout expected a very difficult transition to adult care, compared with 9% of all HCPs surveyed who expected a very difficult transition. Furthermore, HCPs with high burnout expected the transition period to be only 3.2 months compared with 5.3 months overall. Half of HCPs with high burnout (50%) experienced patient reluctance to transition to adult care compared with 33% overall.

HCPs with some burnout (n = 79) were characterized by 1 of 2 factors: they either lacked belief that their work was meaningful (only 13% of HCPs with some burnout strongly believed that their work is meaningful vs 46% overall) or they experienced depression for ≥2 days per month, despite believing their work was meaningful.

HCPs with no burnout (n = 53) had a lower number of inpatients per week (<28 patients/wk) vs HCPs with high burnout (≥28 inpatients/week). They also had a strong belief that their work was meaningful and were either epileptologists/neurologists who experienced depression for <2 d/mo, or epileptologists who experienced depression for ≥2 d/mo.

**Figure 3:**
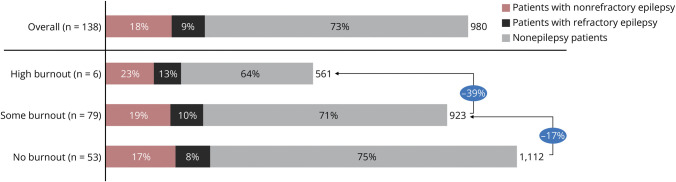
Unique Patient Caseload per Year by Patient Type for Each HCP Burnout Tier and Overall The number at the end of each bar represents the unique patient count per year. The percentages in blue ovals indicate the decrease in unique patient count between burnout tiers. HCP = health care provider.

Unique patient caseload, which was not a major factor affecting burnout, decreased with increasing levels of burnout (Figure 3). However, HCPs with high burnout had a higher yearly caseload of patients with refractory epilepsy (13%) compared with those respondents who indicated no burnout (8%) or some burnout (10%) (Figure 3).

Finally, 32% of HCPs indicated that the likelihood of stopping practicing or moving to another practice within 5 years was probable or very probable (eTable 2, links.lww.com/CPJ/A511). Adult neurologists and pediatric neurologists are the most and least probable to leave practice, respectively. Reasons for leaving practice were not specified.

### Treatment and Management of Refractory vs Nonrefractory Epilepsy

**Figure 4:**
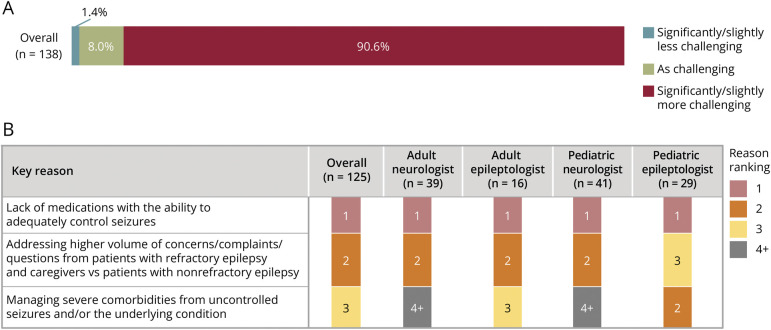
Management of Refractory Epilepsy (A) Proportion of HCPs describing treatment of refractory epilepsy as significantly/slightly less challenging, as challenging, or significantly/slightly more challenging vs nonrefractory epilepsy. (B) Ranked reasons for greater difficulty in managing patients with refractory epilepsy with 1 = most important reason. The top 3 reasons are shown here. HCP = health care provider.

**Figure 5:**
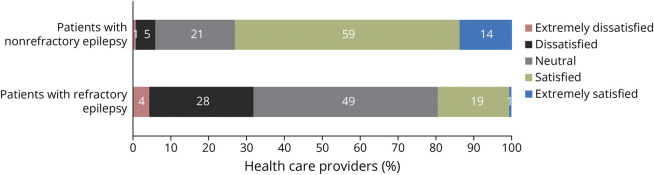
Health Care Provider Satisfaction Level With Available Antiseizure Medications for Patients With Nonrefractory or Refractory Epilepsy More HCPs were at least dissatisfied with available ASMs for refractory epilepsy compared with ASMs for nonrefractory epilepsy. ASM = antiseizure medication; HCP = health care provider.

Approximately 90% of HCPs agreed that treatment for patients with refractory vs nonrefractory epilepsy was more difficult to manage (Figure 4A). Lack of effective medications, the high volume of follow-ups, and treatment of comorbidities make the management of patients with refractory epilepsy more challenging than for patients with nonrefractory epilepsy (Figure 4B). The proportion of HCPs satisfied or extremely satisfied with available ASMs for patients with refractory epilepsy was nearly 4 times less than that for those with nonrefractory epilepsy (20% vs 73%) (Figure 5). Efficacy and safety and tolerability were rated as the 2 most important attributes when selecting an ASM for patients with refractory epilepsy (eFigure 2, links.lww.com/CPJ/A509). HCP dissatisfaction with ASMs when treating patients with refractory epilepsy was mostly due to workload and latency of the insurance approval process, patient out-of-pocket costs, and poor efficacy, safety, and tolerability (eFigure 3, links.lww.com/CPJ/A510).

## Discussion

HCP burnout is a crisis that threatens the quality and accessibility of the US health care system.^23^ The demand for more neurologists is already greater than the number of neurologists currently in practice.^10^ In this study, we conducted a survey among neurologists and epileptologists in the United States and found that 61% of respondents had at least some burnout. Burnout was characterized by HCP caseload of pediatric patients and inpatients, expectations surrounding the transition from pediatric to adult care, number of days physicians experience depression per month, and beliefs regarding meaningfulness of work. Notably, 32% of respondents believed that it was probable or very probable they would stop practicing or move to another practice within the next 5 years. In addition, approximately 90% of HCPs agreed that patients with refractory epilepsy are more challenging to treat than those with nonrefractory epilepsy, and only 20% of HCPs were satisfied or extremely satisfied with currently available ASMs to treat their patients with refractory epilepsy.

Although physician burnout is widely recognized, there is no consensus on the definition of burnout, and existing studies report a range of burnout thresholds and measuring tools.^20,21^ Thus, the prevalence of burnout among physicians remains unknown, with no agreement on a standard measuring tool and contributing factors.^21^ This study evaluated HCP burnout with the MBI-HSS scale, the most frequently used instrument to assess physician burnout levels. In addition, we applied a similar definition of burnout used in several prior studies.^4-6,21^

Results from our study are consistent with those from 2 studies conducted in 2011 and 2014, in which the authors investigated the rate of HCP burnout, how HCP burnout compares with that in the general US workforce, and which HCP specialties have the highest risk of burnout.^4,5^ They found that the proportion of HCPs with burnout is increasing over time and is higher relative to the general US workforce.^4,5^ Furthermore, neurologists are among the specialties with the highest risk of burnout.^4,5^ These findings were supported by a more recent study revealing that 60% of neurologists had at least 1 symptom of burnout.^6^ Similarly, we found that 61% of neurologists/epileptologists who treat patients with refractory epilepsy have at least 1 indicator of burnout.

Previously identified predictors of burnout among HCPs are high caseload per week, nights on call, number of outpatients seen, feelings of loss of autonomy, a high administrative burden, and an inadequate support staff.^6^ This study confirmed that a high caseload per week contributes to burnout; HCPs with high burnout reported more than twice as many inpatients and outpatients compared with the average number overall (180 patients/wk vs 83 patients/wk). Specifically, a high pediatric caseload and increased number of inpatients per week were identified as contributors to HCP burnout. In managing the treatment of pediatric patients, HCPs also require more interactions with caregivers, and a high inpatient caseload can involve more care compared with outpatient care.

Of interest, we found that HCPs with high burnout expected a smooth and quick transition from pediatric to adult care but were surprised by patient reluctance. Transitioning from pediatric to adult care brings several challenges for patients and their caregivers as more independence of the patient is required. Facing the change from an often long-established care team creates hesitancy, and previously nonrelevant topics, such as transportation or career development, come to the forefront. In addition, caregivers might worry that HCPs treating adult patients with epilepsy might not be as experienced with conditions such as DS and LGS due to their childhood onset.^24^ Thus, educating HCPs about the challenges of transitioning patients from pediatric to adult care and providing actionable steps to incorporate into their practice might allow for a smoother process and alleviate some of the stressors. For example, longer duration of follow-up and availability of the pediatric neurologist positively affect patient transition of care.^24^ Finally, we found that a strong belief in the meaning of their work is crucial for HCP well-being, which was similarly reported in other studies.^6^

This study adds to the understanding of how treatment of patients with refractory epilepsy affects HCP burnout. The survey results show that HCPs with high burnout had a higher yearly workload of patients with refractory epilepsy compared with respondents with no or some burnout. Although this result is not statistically significant, likely due to low numbers of patients with refractory epilepsy, this trend indicates that the special challenges associated with treating these patients negatively affect HCPs' well-being. The lack of medications that can adequately control seizures was rated as the top challenge when treating patients with refractory epilepsy. Only 20% of HCPs were satisfied or extremely satisfied with the currently available ASMs to treat refractory epilepsy. This response signals a high unmet need for more effective ASMs, which in turn may decrease levels of HCP burnout.^19,25,26^

Our study has several limitations. We did not assess how access to surgery for epilepsy or epilepsy monitoring, beyond ASM safety monitoring, may affect HCP burnout. Furthermore, evidence from our study suggests that burnout is high for neurologists and clinicians who treat patients with refractory epilepsy. In addition, previous literature shows that neurologists have higher levels of burnout compared with other HCPs. Therefore, our inclusion criteria (neurologists treating ≥10 unique patients with refractory epilepsy per year) may have selected for a burnout-enriched population.^4-6^ The small sample size of pretest participants might also have introduced bias. The overall survey sample size (N = 138) is a limited representation of the US neurologist population who treat patients with refractory epilepsy. The number of physicians captured in the high burnout tier is also limited (n = 6), affecting the accuracy of the statistical analysis and drivers' output. Bias was limited by normalizing the responses for each respondent and reporting the mean, median, and SD on the normalized scores; nevertheless, information bias, selection bias, or bias linked to inconsistent use of the rating scales between respondents may have influenced the results of this study. Although recursive partitioning is a comprehensive machine learning algorithm, combinations of local best choices at each decision tree node may not guarantee a globally optimal solution. Finally, the chosen definition of high burnout does not consider that HCPs with a high score in any 1 domain might experience an impactful burnout syndrome similar to HCPs with high scores in all 3 domains.

Nonetheless, our study provides valuable insights into burnout in neurologists who treat patients with refractory epilepsy and to identify challenges associated with managing those patients. We chose the well-established MBI-HSS scale to assess burnout levels and included pilot testing to improve reliability of the results.

In summary, some level of burnout is common among HCPs who treat patients with refractory epilepsy. High burnout is driven by both high pediatric and inpatient caseloads and by difficulty with transitioning pediatric patients to adult care. Some burnout is linked to HCPs' lack of belief that their work is meaningful, the number of days per month they experience depression, and specialty. Although burnout is not driven by unique patient load, there is a trend toward neurologists and epileptologists experiencing higher levels of burnout with an increase in the caseload of patients with refractory epilepsy. It is probable or very probable that more than one-third of neurologists/epileptologists will stop practicing or move to another practice within 5 years. Efficacy and safety and tolerability of ASMs are considered the most important attributes for HCP satisfaction with available ASMs; however, levels of current satisfaction with those attributes and overall satisfaction with ASMs to treat patients with refractory epilepsy are low. Thus, there is a critical unmet need for safe and effective ASMs for the treatment of patients with refractory epilepsy. Further studies can inform whether access to improved treatment options for patients with refractory epilepsy might alleviate physicians' burnout.

## Appendix Authors

**Table:**

Name	Location	Contribution
Mei Lu, MD, MS	Takeda Pharmaceuticals USA, Inc, Lexington, MA	Drafting/revision of the article for content, including medical writing for content; major role in the acquisition of data; and study concept or design
Enrico Speri, PhD	IQVIA, Cambridge, MA	Drafting/revision of the article for content, including medical writing for content; major role in the acquisition of data; study concept or design; and analysis or interpretation of data
A. Lauks, MSc	IQVIA, Cambridge, MA	Drafting/revision of the article for content, including medical writing for content; major role in the acquisition of data; study concept or design; and analysis or interpretation of data
Charles Leibson, BA	IQVIA, Cambridge, MA	Drafting/revision of the article for content, including medical writing for content; major role in the acquisition of data; study concept or design; and analysis or interpretation of data
Satish C. Rao, MD, MS	Takeda Pharmaceuticals USA, Inc., Lexington, MA	Drafting/revision of the article for content, including medical writing for content; major role in the acquisition of data; and study concept or design
